# Phylogenetic analysis of *Haemaphysalis erinacei* Pavesi, 1884 (Acari: Ixodidae) from China, Turkey, Italy and Romania

**DOI:** 10.1186/s13071-016-1927-1

**Published:** 2016-12-15

**Authors:** Sándor Hornok, Yuanzhi Wang, Domenico Otranto, Adem Keskin, Riccardo Paolo Lia, Jenő Kontschán, Nóra Takács, Róbert Farkas, Attila D. Sándor

**Affiliations:** 1 Department of Parasitology and Zoology, University of Veterinary Medicine, Budapest, Hungary; 2 Department of Pathogenic Biology, School of Medicine, Shihezi University, Shihezi, China; 3 Department of Animal Health and Welfare of the Faculty of Veterinary Medicine, University of Bari, Bari, Italy; 4 Department of Biology, Gaziosmanpasa University, Tasliciftlik-Tokat, Turkey; 5 Plant Protection Institute, Centre for Agricultural Research, Hungarian Academy of Sciences, Budapest, Hungary; 6 Department of Parasitology and Parasitic Diseases, University of Agricultural Sciences and Veterinary Medicine, Cluj-Napoca, Romania

**Keywords:** Tick, *Haemaphysalis*, *cox*1 gene, 16S rRNA gene, Phylogeography, Phylogenetics

## Abstract

**Background:**

*Haemaphysalis erinacei* is one of the few ixodid tick species for which valid names of subspecies exist. Despite their disputed taxonomic status in the literature, these subspecies have not yet been compared with molecular methods. The aim of the present study was to investigate the phylogenetic relationships of *H. erinacei* subspecies, in the context of the first finding of this tick species in Romania.

**Results:**

After morphological identification, DNA was extracted from five adults of *H. e. taurica* (from Romania and Turkey), four adults of *H. e. erinacei* (from Italy) and 17 adults of *H. e. turanica* (from China). From these samples fragments of the cytochrome *c* oxidase subunit 1 (*cox*1) and 16S rRNA genes were amplified via PCR and sequenced. Results showed that *cox*1 and 16S rRNA gene sequence divergences between *H. e. taurica* from Romania and *H. e. erinacei* from Italy were below 2%. However, the sequence divergences between *H. e. taurica* from Romania and *H. e. turanica* from China were high (up to 7.3% difference for the 16S rRNA gene), exceeding the reported level of sequence divergence between closely related tick species. At the same time, two adults of *H. e. taurica* from Turkey had higher 16S rRNA gene similarity to *H. e. turanica* from China (up to 97.5%) than to *H. e. taurica* from Romania (96.3%), but phylogenetically clustered more closely to *H. e. taurica* than to *H. e. turanica*.

**Conclusions:**

This is the first finding of *H. erinacei* in Romania, and the first (although preliminary) phylogenetic comparison of *H. erinacei* subspecies. Phylogenetic analyses did not support that the three *H. erinacei* subspecies evaluated here are of equal taxonomic rank, because the genetic divergence between *H. e. turanica* from China and *H. e. taurica* from Romania exceeded the usual level of sequence divergence between closely related tick species, suggesting that they might represent different species. Therefore, the taxonomic status of the subspecies of *H. erinacei* needs to be revised based on a larger number of specimens collected throughout its geographical range.

## Background

**Fig. 1:**
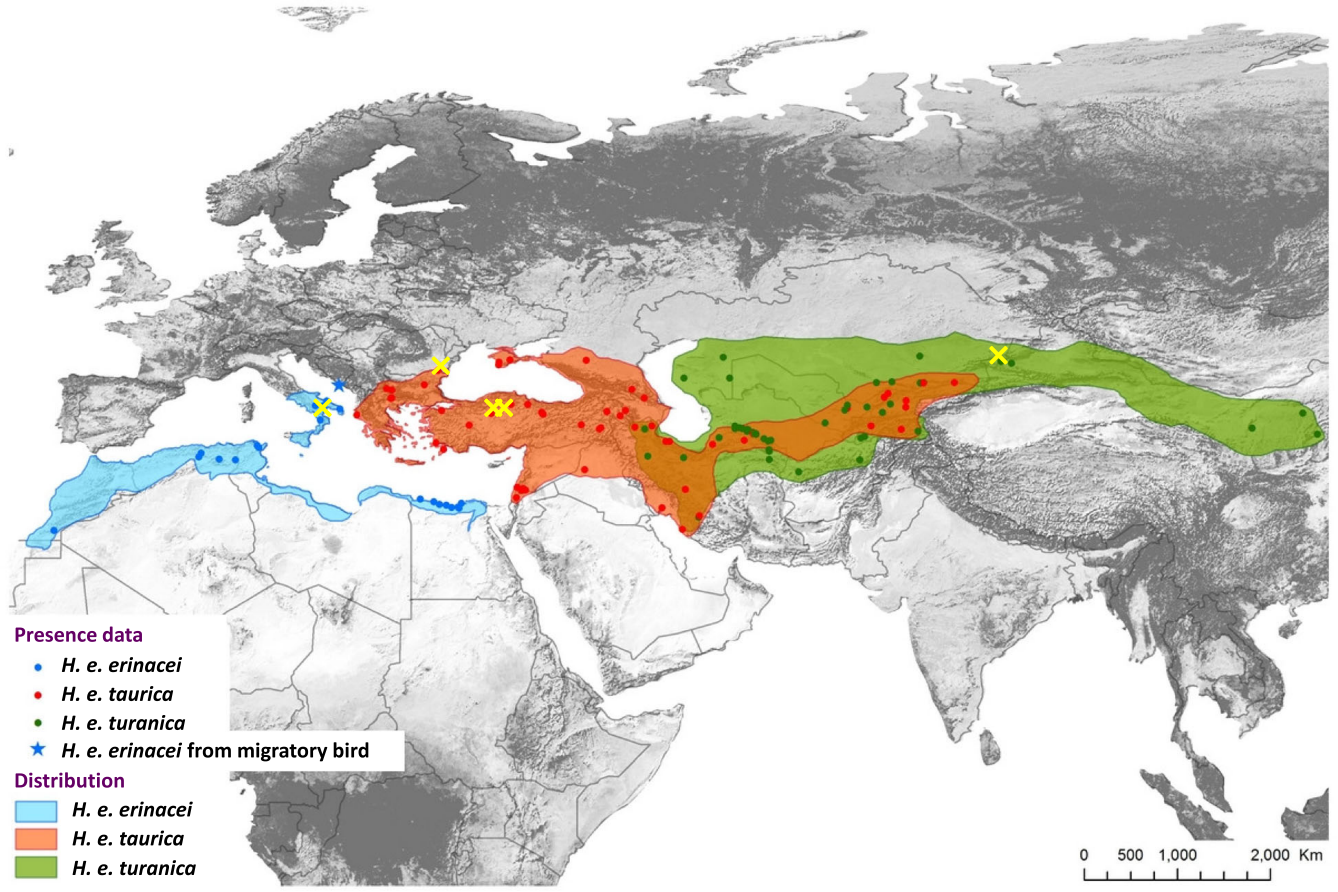
Distribution map of the three subspecies of *Haemaphysalis erinacei* based on literature data, and including geographical locations of the specimens collected in the present study (*yellow crosses*)

*Haemaphysalis* Koch, 1844 is the second largest genus (following *Ixodes*) of hard ticks (Acari: Ixodidae), including 167 species [1]. Among them, *Haemaphysalis erinacei* Pavesi, 1884 occurs in Mediterranean forests, woodlands and scrub [1], with a geographical range covering Central Asia (including Afghanistan, Pakistan and Western China), Crimea, the Middle East, southern Europe and North Africa (Fig. 1). The preferred hosts of *H. erinacei* are terrestrial mammals, such as hedgehogs and carnivores for adult ticks [1] and rodents mainly for larvae and nymphs [1–3]. Bats, birds and reptiles are considered as accidental hosts [4–6]. This species is also known to feed on humans in the adult stage [7], and is a potential vector of zoonotic rickettsiae (*Rickettsia massiliae* [8], *R. raoultii* [9] and *R. heilongjiangensis* [10]).

*Haemaphysalis erinacei* is one of the few ixodid tick species for which valid names of subspecies exist. Subspecies are conspecific taxa, representatives of which show differences in morphology and geographical range from each other, but can naturally interbreed. Accordingly, until now *H. erinacei* subspecies were described on the basis of different morphology and geographical range, but this resulted in a controversy in their taxonomy. Camicas et al. [11] listed four valid subspecies of *H. erinacei*, namely *H. e. erinacei* Pavesi, 1884 (described from Tunisia), *H. e. ornata* Feldman-Muhsam, 1956 (described from Israel), *H. e. taurica* Pospelova-Shtrom, 1940 (described from Crimea) and *H. e. turanica* Pospelova-Shtrom, 1940 (described from Tajikistan). According to Hoogstraal [12] *H. erinacei* includes three subspecies: *H. e. erinacei* in North Africa, *H. e. taurica* in the Middle East (including western states of the former Soviet Union) and *H. e. turanica* in Central Asia. *Haemaphysalis e. erinacei* also occurs in southern Europe, in particular in Spain, Italy and the western Balkans [4], whereas *H. e. taurica* is present in the eastern Balkans [6], Crimea and the Caucasus (i.e. near the eastern Balkans and the Middle East); both *H. e. taurica* and *H. e. turanica* are widely distributed in certain regions of Central Asia (Fig. 1).

Despite this taxonomic controversy, no studies have attempted molecular phylogenetic comparison of *H. erinacei* subspecies. Based on the above literature data on their morphology and geographical range, we hypothesized that phylogenetic analyses would support *H. e. turanica* as a separate species from *H. e. taurica* and *H. e. erinacei*. Therefore, in the present study the phylogenetic relationships of *H. erinacei* subspecies (collected in four countries) were investigated, in the context of the first finding of this tick species in Romania.

Cytochrome *c* oxidase subunit 1 (*cox*1) and 16S rDNA genes are well-established barcoding genes for molecular identification and phylogenetic analyses of ticks [13–16]. Therefore analysis of these two genes was chosen to investigate the phylogenetic relationships of *H. e. taurica*, *H. e. erinacei* and *H. e. turanica* in the present study.

## Methods

### Sample origin and morphological analysis

Altogether 26 adults of *H. erinacei* were included in this study (Table 1). The subspecies were identified according to Hoogstraal [3] (*H. e. erinacei*) and Filippova [17] (*H. e. turanica*, *H. e. taurica*). Pictures were produced with a VHX-5000 (Keyence Co., Osaka, Japan) digital microscope.

**Table 1:** Data for *Haemaphysalis erinacei* used in this study. The sex/stage of ticks and date of collection are not shown

*H. erinacei* subspecies	Country	Location	Origin	*cox*1 sequence similarity with (*)	*cox*1 sequence ID (isolate, *n* = number > 1)	16S sequence similarity with (*)	16S sequence ID (isolate)
*H. e. turanica*	China	Alataw Pass	Vormela peregusna	605/636 bp (95.1%)	KU880621 (ABL1)	355/374 bp (94.9%)	KU880549 (ABL1)
		Alataw Pass		605/636 bp (95.1%)	KU880609 (ABL6)	–	–
		Alataw Pass		603/636 bp (94.8%)	KU880620 (ABL5-3)	–	–
		Alataw Pass		603/636 bp (94.8%)	KU880608 (ABL5-1)	–	–
		Alataw Pass		604/636 bp (95.0%)	KU880607 (ABL2)	383/406 bp (94.3%)	KU880556 (ABL2)
		Alataw Pass		604/636 bp (95.0%)	KU880559 (ABL5)	–	–
		Alataw Pass		577/608 bp (94.9%)	KU880616 (ABL5-2)	–	–
		Alataw Pass		575/608 bp (94.6%)	KU880615 (ABL4)	352/374 bp (94.1%)	KU880550 (ABL4)
		Alataw Pass		605/636 bp (95.1%)	KU880589 (ALSK186-1)	–	–
		Alataw Pass		604/636 bp (95.0%)	KU880573 (ALSK186)	351/374 bp (93.9%)	KU880551 (ALSK186)
		Alataw Pass		603/636 bp (94.8%)	KU880572 (ALSK185)	–	–
		Alataw Pass		–	–	384/405 bp (94.8%)	KU880555 (ABL1-1)
		Alataw Pass		–	–	386/405 bp (95.3%)	KU880557 (ABL10)
		Alashankou		–	–	356/384 bp (92.7%)	KR053302 (1)
		Alashankou		–	–	361/382 bp (94.5%)	KR053303 (2)
		Alashankou		–	–	362/382 bp (94.8%)	KR053304 (3)
		Alashankou		–	–	361/383 bp (94.3%)	KR053305 (4)
H. e. taurica*	Romania	Canaraua Fetii	cave entrance	636/636 bp (100%)	KU885986	404/404 (100%)	KU885987
*H. e. taurica*	Turkey	Tokat Province	*Homo sapiens*	636/636 bp	KX901844 (*n* = 2)	401/404 bp (99.3%)	KX901845
		Sivas Province	*Erinaceus concolor*	not successful	– (*n* = 2)	391/406 bp (96.3%)	KX901846
*H. e. erinacei*	Italy	Basilicata region	*Martes foina*	632/636 bp (99.4%)	KX237631 (*n* = 3)	397/404 bp (98.3%)	KX237632
				632/636 bp (99.4%)	KX237631	397/405 bp (98.0%)	KX237633

*Abbreviations*: *ID* GenBank accession number, *bp* base pairs

*The sample from Romania was the source of the reference sequence

### Molecular analysis

DNA was extracted with QIAamp DNA Mini Kit (QIAGEN, Hilden Germany) as described [18], including an overnight digestion step at 56 °C in tissue lysis buffer and 6.6% proteinase-K (provided by the manufacturer). Two mitochondrial markers were amplified from selected samples: an approx. 710 bp long fragment of the cytochrome *c* oxidase subunit 1 (*cox*1) gene using the primers HCO2198 (5′-TAA ACT TCA GGG TGA CCA AAA AAT CA-3') and LCO1490 (5′-GGT CAA CAA ATC ATA AAG ATA TTG G-3′) [19], and an approx. 460 bp fragment of the 16S rDNA gene using the primers 16S + 1 (5′-CTG CTC AAT GAT TTT TTA AAT TGC TGT GG-3′) and 16S-1 (5′-CCG GTC TGA ACT CAG ATC AAG T-3′) [13]. Reaction conditions were set as reported [20]. Concerning samples collected in China, another set of primers (designed by Primer Premier 5.0 software) was used for the *cox*1 gene (forward: 5′-ATT TAC AGT TTA TCG CCT-3′; reverse: 5′-CAT ACA ATA AAG CCT AAT A-3′), and PCR conditions were different (preheating at 94 °C for 4 min, followed by 40 cycles of denaturation at 94 °C for 30 s, annealing at 45 °C for 1 min and extension at 72 °C for 1 min; final elongation at 72 °C for 8 min).

PCR products were visualized in 1.5% agarose gel. Purification and Sanger dideoxy sequencing for samples from Romania, Italy and Turkey was done by Biomi Inc. (Gödöllő, Hungary), and for samples from China by Sangon Biotech Co. (Shanghai, China). The newly-generated sequences were manually edited, aligned and compared to reference GenBank sequences by nucleotide BLASTN program (https://blast.ncbi.nlm.nih.gov). Representative sequences were submitted to the GenBank database (Table 1). The automatic MEGA model selection method (analysis: Maximum Likelihood model selection, substitution type: nucleotide) was applied to choose the appropriate model for phylogenetic analyses. The dataset was resampled 1000 times to generate bootstrap values. Phylogenetic analyses were conducted with the Maximum Likelihood method (HKY model [21]) by using MEGA version 6.0 [22]. Outgroups of phylogenetic trees were selected from GenBank (from ixodid genera other than *Haemaphysalis*), and are referenced according to accession numbers.

## Results and discussion

The *cox*1 nucleotide sequence of *H. e. taurica* from Romania was 100% identical with the sequence for the same subspecies from Turkey (Tokat province), and 99.4% identical with *H. e. erinacei* from Italy, but had only 94.6–95.1% similarity with isolates of *H. e. turanica* from China (Table 1). Concerning the amplified part of the 16S rRNA gene, *H. e. taurica* from Romania showed 99.3% similarity with one specimen of *H. e. taurica* collected in Turkey (Tokat province), and 98–98.3% similarity with *H. e. erinacei* from Italy. On the other hand, the 16S rRNA fragment of *H. e. taurica* from Romania had only 92.7–95.3% similarity with isolates of *H. e. turanica* from China.

Taken together, the *cox*1 and 16S rDNA gene sequence divergences between *H. e. taurica* from Romania and Turkey (Tokat province) and *H. e. erinacei* from Italy were low (below 2%). This may be consistent with allopatric separation of these two subspecies (Fig. 1). Similar magnitudes of intraspecific genetic (i.e. 1.2%) variation in the 16S rRNA target region have been recorded for other ixodid species, such as *I. scapularis*, over large geographical distances [15]. However, the sequence divergence between *H. e. taurica* from Romania and *H. e. turanica* from Central-Asia was high (up to 5.4% for the *cox*1 gene, and up to 7.3% for the 16S rRNA gene; Table 1), i.e. exceeding the expected (average) level of sequence divergence between closely related tick species [14]. For comparison, the 16S rRNA gene sequence similarity between *I. inopinatus* (KM211790) and *I. ricinus* (GU074592) is 98.2% (383/390 bp), amounting to 1.8% difference [16]. When species boundaries were evaluated for several tick species [14], the sequence divergence delineating tick species was reported to be 5.3% for the 16S rRNA gene, i.e. much lower than the 7.3% shown here for *H. e. taurica* and *H. e. turanica*.

**Fig. 2:**
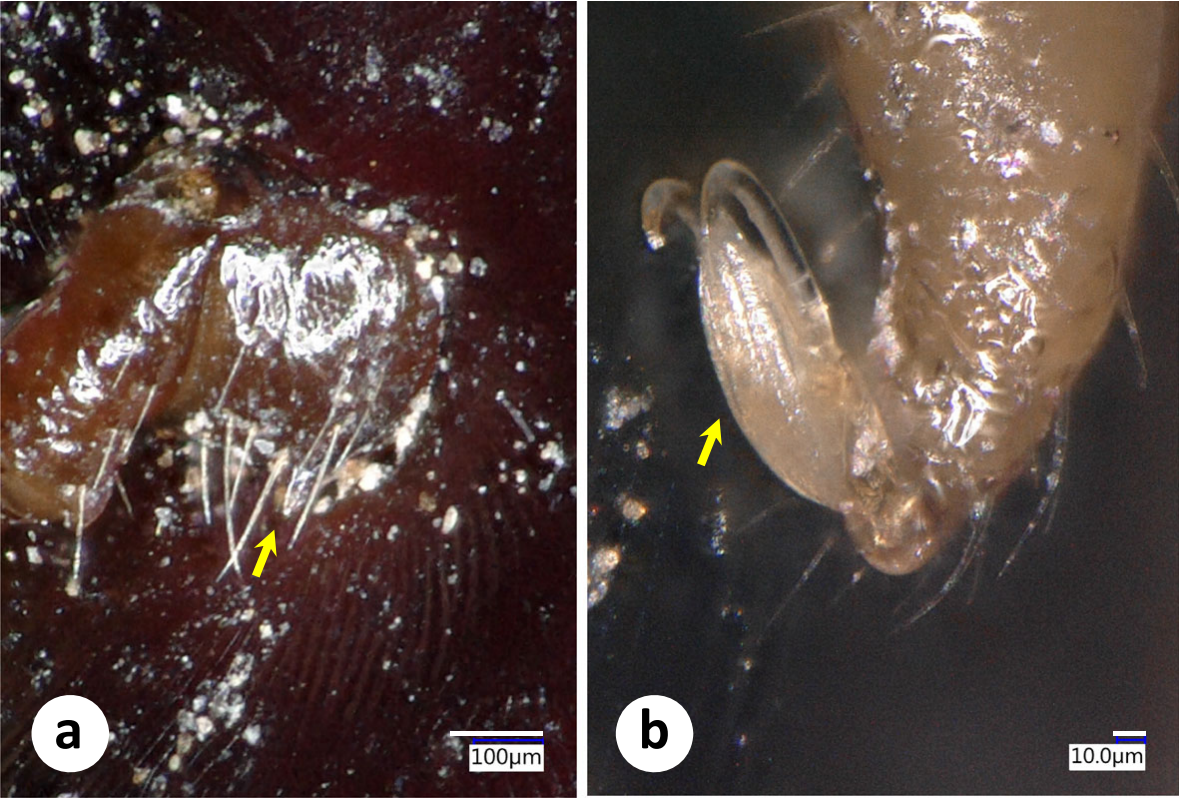
Morphology of genetically divergent *H. e. taurica* female from Turkey (Sivas Province) identified according to Filippova [17]. **a** Caudolateral setae on coxa IV are much longer than the spur (*arrow*). **b** The pulvillus (*arrow*) almost reaches the ends of claws

Interestingly, two females from Turkey (Sivas province), which were morphologically identified as adults of *H. e. taurica* (Fig. 2) had higher 16S rRNA gene similarity to isolates of *H. e. turanica* from China (maximum 396/406 bp = 97.5%) than to *H. e. taurica* from Romania (96.3%) (Table 1). However, these two samples clustered phylogenetically more closely to *H. e. taurica* than to *H. e. turanica* (Fig. 4), indicating the existence of different genetic lineages within *H. e. taurica*.

**Fig. 3:**
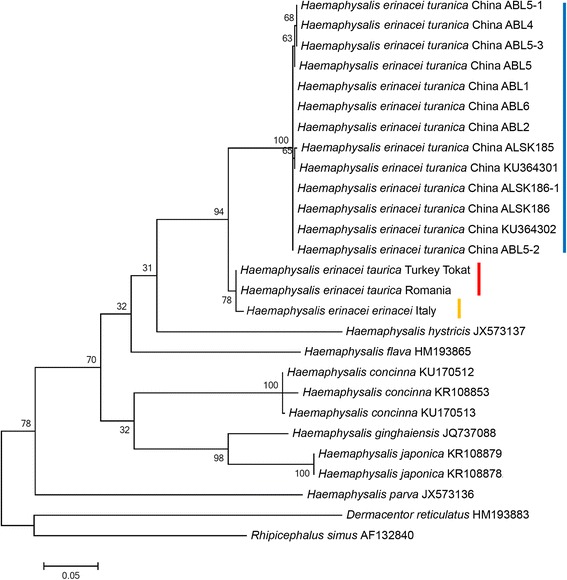
Phylogenetic relationships of *Haemaphysalis* spp., including *H. erinacei* ssp., based on the amplified part of the *cox*1 gene. Representative genotypes of ticks from this study are marked with location and isolate code (see Table 1 for details). The vertical *red*, *yellow* and *blue* lines mark the *H. e. taurica*, *H. e. erinacei* and *H. e. turanica* clades, respectively. Branch lengths represent the number of substitutions per site inferred according to the scale shown

**Fig. 4:**
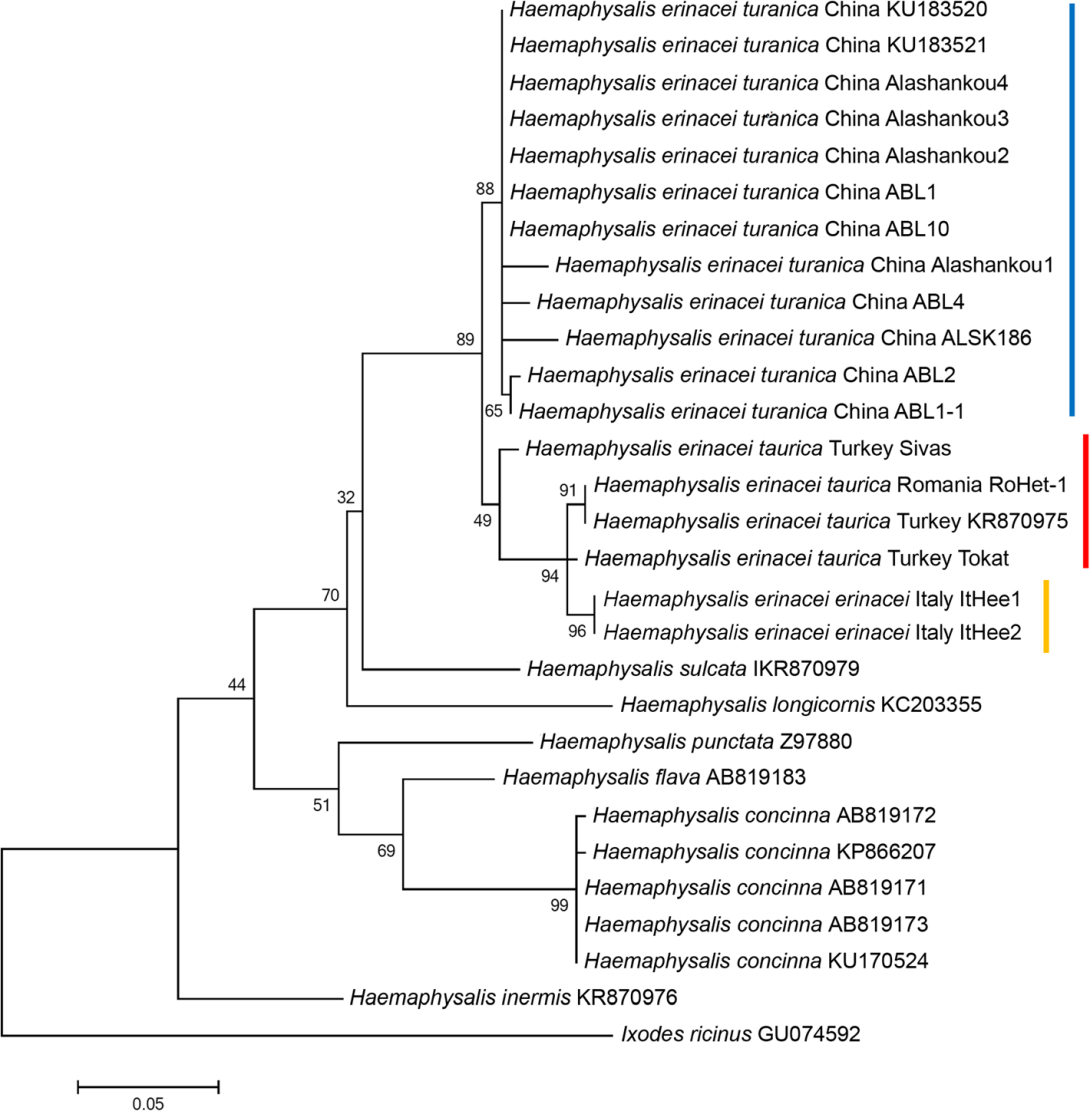
Phylogenetic comparison of 16S rDNA sequences of *Haemaphysalis* spp., including *H. erinacei* ssp. Representative genotypes of ticks from this study are marked with location and isolate code (see Table 1 for details). The vertical *red*, *yellow* and *blue* lines mark the *H. e. taurica*, *H. e. erinacei* and *H. e. turanica* clades, respectively. Branch lengths represent the number of substitutions per site inferred according to the scale shown

The number/percentage of nucleotide differences between *H. e. taurica*, *H. e. erinacei* and *H. e. turanica* are well reflected by the topology of *cox*1 phylogenetic tree, with *H. e. taurica* and *H. e. erinacei* clustering close to each other, but separately from *H. e. turanica* (Fig. 3). This separation was supported by a high probability (94%), and chronologically (based on branch lengths) preceded the separation of *H. e. taurica* and *H. e. erinacei* (Fig. 3). The phylogenetic analysis of 16S rRNA gene sequences confirmed these relationships, i.e. all genotypes of *H. e. turanica* clustered in one clade, as a sister group to all *H. e. erinacei* and *H. e. taurica* isolates (Fig. 4).

In a geochronological context, the divergence of *H. punctata* and *H. flava* was estimated to have taken place approx. 40 million years ago [23]. Relative to this event, as inferred from the branch lengths in the 16S rRNA gene phylogenetic tree (Fig. 4), the divergence of *H. e. turanica* from *H. e. taurica*/*erinacei* might have occurred much more recently.

Several factors may have contributed to this divergence and its maintenance. Southern peninsulas of Europe acted as major refugia during ice age(s), from which genetically distinct clades of animal species emerged [24], as also exemplified by *H. e. erinacei* and *H. e. taurica*. Similarly, glacial surfaces confluent with the Caspian Sea [25] may have caused east-to-west separation of *H. e. taurica* and *H. e. turanica*. Consequently, frequent genetic mixing between the latter populations might have been inhibited by at least two factors. First, birds are only accidental hosts of *H. erinacei* [4], whereas its typical hosts (i.e. hedgehogs) do not migrate, preventing genetic mixing over large distances. For comparison, *Haemaphysalis* spp. frequently infesting birds show minimal or no *cox*1 or 16S rRNA gene heterogeneity over very large geographical distances: e.g. *H. concinna* [26]; or *H. punctata* from Spain (Z97880), Turkey (KR870978) and China (KF547980) with 100% 16S rRNA gene identity.

In addition, based on an extensive collection material, while the hosts of *H. e. taurica* and *H. e. turanica* are common, these two tick subspecies exhibit biotope isolation in overlapping parts of their geographical range [17], which most likely reduced further the chances of gene flow between their populations.

## Conclusions

This is the first finding of *H. erinacei* in Romania, and the first (although preliminary) phylogenetic comparison of *H. erinacei* subspecies. Phylogenetic analyses do not support that the three *H. erinacei* subspecies evaluated here are of equal taxonomic rank. In particular, the genetic divergence between *H. e. turanica* from China and *H. e. taurica* from Romania exceeded the usual level of sequence divergence between closely related tick species, suggesting (especially if formerly reported morphological differences are also taken into account) that they might represent different species. Therefore, the taxonomic status of the subspecies of *H. erinacei* needs to be revised based on a larger number of specimens collected throughout its geographical range.
